# Willingness to accept Doxycycline post-exposure prophylaxis for bacterial stis prevention among men who have sex with men in Southern China: a cross-sectional analysis

**DOI:** 10.1186/s12879-025-11290-x

**Published:** 2025-07-11

**Authors:** Peng Liang, Peizhen Zhao, Shujie Huang, Cheng Wang

**Affiliations:** 1 Dermatology Hospital, Southern Medical University, Guangzhou, 510095 China; 2 Southern Medical University Institute for Global Health, Guangzhou, 510095 China

**Keywords:** Men who have sex with men, Doxycycline, Prophylaxis, Doxy-PEP, Sexually transmitted infections, China

## Abstract

**Background:**

Doxycycline post-exposure prophylaxis (doxy-PEP) has the potential to decrease the incidence of sexually transmitted infections (STIs) among men who have sex with men (MSM). However, there is a limited literature on willingness to use doxy-PEP among Chinese MSM. This study aimed to examine the willingness to use doxy-PEP and its associated factors among MSM in China.

**Methods:**

In 2023, we conducted a cross-sectional study in four cities in Guangdong provinces in South China. Data were collected on social-demographic characteristics, sexual behaviors, and the willingness to use doxy-PEP. Multivariable logistic regression analysis was employed to identify factors associated with the willingness to use doxy-PEP.

**Results:**

A total of 884 individuals were included in this study. Of these, 53.5% reported a willingness to use doxy-PEP for the prevention of STIs. The main concerns for those unwilling to use doxy-PEP were related to its efficacy (82.5%) and safety (81.7%). Multivariable logistic regression indicated that participants were more likely to express willingness to use doxy-PEP if they had female sexual partners in the past 6 months (adjusted Odds Ratio (aOR): 1.58, 95% CI: 1.11–2.25), had prior or current doxy-PEP use (aOR = 2.39, 95% CI: 1.13–5.05), or had peers currently using doxy-PEP (aOR = 1.58, 95% CI: 1.09–2.30).

**Conclusions:**

The willingness to use doxy-PEP among MSM in South China is relatively low. These findings highlight the need for effective educational and targeted intervention strategies to raise awareness and knowledge toward using doxy-PEP among MSM in China.

**Supplementary Information:**

The online version contains supplementary material available at 10.1186/s12879-025-11290-x.

## Background

Bacterial sexually transmitted infections (STIs), including syphilis, gonorrhea, and chlamydia, continue to pose a significant public health threat, especially among men who have sex with men (MSM) [1]. Among Chinese MSM, reported prevalence rates are 12% for syphilis [2], 6.5% for chlamydia [3], and 2.5% for gonorrhea [4]. Despite interventions such as sex education, free condom distribution, and regular STIs screening [5, 6], these measures have not sufficiently contained the spread of STIs [7, 8].

Recently, Doxycycline post-exposure prophylaxis (doxy-PEP) has emerged as a promising biomedical approach to prevent chlamydia and syphilis in MSM. Randomized controlled trials in high-income countries, including France [9] and the United States [10], indicate that a single dose of 200 mg Doxycycline taken within 72 h of unprotected sexual contact significantly reduces syphilis and chlamydia infections among MSM and transgender women. Reflecting these findings, the U.S. Centers for Disease Control and Prevention (CDC) issued provisional guidelines recommending doxy-PEP for MSM at elevated STIs risk [11]. Real-world data from San Francisco support this recommendation, showing a 78% decrease in syphilis cases and a 67% decrease in chlamydia cases within the first 13 months of doxy-PEP adoption [12].

To enable effective implementation in China, understanding the willingness of MSM to use doxy-PEP is crucial. However, data on doxy-PEP willingness among Chinese MSM are scarce; only one cross-sectional study in three Chinese cities (Nanjing, Wuhan and Changsha) has found a 75% willingness to use doxy-PEP for syphilis prevention [13]. This study thus aimed to assess MSM willingness to use doxy-PEP for STIs prevention and examine related factors among MSM in South China.

## Methods

### Study site

A cross-sectional survey was conducted from April to August 2023 in Guangdong Province, Southern China, with a focus on the cities of Yunfu, Foshan, Jiangmen, and Shenzhen. Guangdong, China’ s wealthiest province and a global economic center, consists of 21 municipalities with pronounced economic disparities; GDP per capita in the richest areas is up to eight times higher than in the least developed regions [14]. This economic variation allowed representative sampling across diverse socioeconomic levels. Additionally, Guangdong has ranked consistently among the top three provinces for STIs incidence over the past decade [15, 16], making it an essential area for STIs research and providing a strong foundation for this study.

### Participant recruitment

At each site, MSM outreach teams were assembled, including nurses, clinical physicians, and public health experts. Drawing on extensive outreach experience, these teams provided syphilis testing, condom distribution, sexual health education, and risk-reduction interventions. Participants were recruited through convenience sampling by local outreach teams in offline settings such as pubs, discos, tearooms, and clubs. Eligibility criteria required that participants be biologically male, over 18 years of age, and have engaged in oral or anal sex with another male within the past year.

### Data collection

Data were collected using a paper-based questionnaire (Supplementary file 1), developed through extensive literature reviews and expert consultation in STIs research. A pilot survey was conducted before the main study to evaluate the questionnaire’s reliability and validity, including internal consistency and test-retest reliability; data from the pilot were excluded from the final analysis. All eligible participants completed the questionnaire, with outreach staff providing verbal support for those needing assistance. Confidentiality and anonymity were ensured through secure data handling, and written informed consent was obtained from each participant.

After completing the questionnaire, participants were instructed to self-collect urine samples, while staff collected blood, anal, and oral swabs at each outreach service site. Urine samples were tested for chlamydia and gonorrhea. Anal swabs were collected from individuals reporting receptive anal sex, and oral swabs from those engaged in insertive oral sex in the past six months, irrespective of condom use. All urine, oral and anal swabs were analyzed at Southern Medical University Dermatology Hospital using Cobas 4800 CT/NG detection kits (Roche Molecular Systems, Inc., New Jersey, USA). Blood samples were tested at local STIs clinics: HIV testing used enzyme-linked immunoassay (ELISA, Lizhu Biotech Inc., Zhuhai, China), and syphilis testing included the rapid plasma reagin (RPR) and Treponema pallidum particle agglutination (TPPA) tests (Rongsheng Biotech Inc., Shanghai, China). For HIV-positive results, the outreach team checked if participants already knew their status to identify new diagnoses. Newly diagnosed cases were referred to the local CDC for confirmatory testing. Individuals were considered syphilis positive if they tested positive for both RPR and TPPA. Positive results were communicated to participants via text within two weeks, and local outreach teams arranged referrals to ensure timely treatment. Post-test counseling and necessary STI/HIV care were provided according to standard clinic protocols.

### Measures

#### Sociodemographic and sexual behavior characteristics

Collected sociodemographic variables included age, ethnicity, marital status, residence, education level, and current employment duration. Sexual behavior variables included the weekly average of anal sex encounters with males, consistency of condom use during anal sex in the past six months, condom use in the most recent anal sex encounter, engagement in commercial sex with males in the past six months, and use of online platforms to find sexual partners. STIs variables included history of STIs diagnoses and current test results. Consistent condom use was defined as always using condoms during anal sex with males. STIs prevalence was determined by either a positive test result or a previous diagnosis of syphilis, chlamydia, or gonorrhea.

#### Awareness and willingness to use Doxycycline post-exposure prophylaxis

Awareness of doxy-PEP was assessed by asking participants if they had heard of doxy-PEP for STIs, if they had ever received or were currently receiving doxy-PEP, and if anyone in their social network had used or was using doxy-PEP. Before assessing willingness, we provided all participants with a brief introduction to doxy-PEP in the questionnaire, which explained its concept and efficacy for STIs prevention. This ensured all participants had basic information about doxy-PEP regardless of their prior awareness. Following this introduction, willingness to use doxy-PEP for STIs prevention was gauged by asking, “Are you willing to use doxy-PEP for STIs prevention?“.

#### Statistical analysis

Descriptive statistics were used to summarize sociodemographic characteristics, sexual behaviors, STIs testing, and doxy-PEP awareness and willingness. Continuous variables were reported as means ± standard deviations (x̄ ± SD) and median, as well as categorical variables as frequencies and percentages. Chi-square tests assessed differences in categorical variables across groups.

Univariable and multivariable logistic regression analyses were conducted to identify factors associated with willingness to use doxy-PEP. Multivariable models included covariates such as age, ethnicity, marital status, educational level, household registration status, and duration of employment at the current location, based on prior literature [13, 17–19]. Results were presented as odds ratios (OR), adjusted odds ratios (aOR), 95% confidence intervals (CI), and P-values, with statistical significance set at *P* ≤ 0.05. All analyses were performed using R (version 4.3.0).

## Results

### Sociodemographic characteristics

Of the 932 eligible participants, 48 declined, leaving a final sample of 884 MSM who completed the survey. The average age was 36.2 ± 12.0 years, with 56.2% aged 35–45 and 43.0% holding at least a college degree. Most were unmarried or divorced (73.9%), had been employed locally for over a year (78.7%), and identified as gay (66.3%) (Table 1).

**Table 1:** Sociodemographic and sexual behavior characteristics of participants using Doxycycline post-exposure prophylaxis (*N* = 884)

Characteristics	Total *n* (%)	Willingness to use doxy-PEP		*P*-value
		No *n* (%)	Yes *n* (%)	
**Demographics**	884	411(46.5)	473(53.5)	
**Age**				0.070
Mean ± SD	36.2 ± 11.6	37.0 ± 12.8	35.5 ± 11.1	
Median (quartiles)	33.0 (27.0–45.0)	34.0 (27.0–47.0)	33.0 (27.0–43.0)	
18–25	174(19.7)	84(20.4)	90(19.0)	
26–35	320(36.2)	131(31.9)	189(40.0)	
36–45	177(20.0)	85(20.7)	92(19.5)	
>45	213(24.1)	111(27.0)	102(21.6)	
**Ethnicity**				0.934
Han	833(94.2)	387(94.2)	446(94.3)	
Non-Han	51(5.8)	24(5.8)	27(5.7)	
**Marital status**				0.187
Unmarried/Divorce	653(73.9)	295(71.8)	358(75.7)	
Married	231(26.1)	116(28.2)	115(24.3)	
**Place of residence**				0.144
Local province	567(64.1)	274(66.7)	293(61.9)	
Other provinces	317(35.9)	137(33.3)	180(38.1)	
**Educational attainment**				0.026*
Junior high school or below	183(20.7)	87(21.2)	96(20.3)	
High school	321(36.3)	166(40.4)	155(32.8)	
College degree or above	380(43.0)	158(38.4)	222(46.9)	
**Duration of employment at the current location**				0.389
< 6 months	117(13.2)	61(14.8)	56(11.8)	
6–12 months	71(8.0)	34(8.3)	37(7.8)	
Over 1 year	696(78.7)	316(76.9)	380(80.3)	
**Sexual orientation**				0.185
Homosexual	586(66.3)	281(68.4)	305(64.5)	
Bisexual	222(25.1)	102(24.8)	120(25.4)	
Other	76(8.6)	28(6.8)	48(10.1)	
**Received any of the AIDS prevention services in the past year**				0.162
No	34(17.2)	20(4.9)	14(3.0)	
Yes	850(82.8)	391(95.1)	850(97.0)	
**Sexual behavior**				
**Number of anal sex with male in the past week**				0.002*
Mean ± SD	1.2 ± 1.6	1.5 ± 1.6	0.9 ± 1.5	
Median (quartiles)	1.0 (0.0–2.0)	1.0 (0.0–3.0)	0.0 (0.0–1.0)	
None	419(47.4)	174(42.3)	245(51.8)	
1–3	405(45.8)	199(48.4)	206(43.6)	
>3	60(6.8)	38(9.2)	22(4.7)	
**Providing anal sex for males in the past 6 months**				0.264
No	56(6.3)	22(5.4)	34(7.2)	
Yes	828(93.7)	389(94.6)	439(92.8)	
**Consistency of condom use during anal sex with male in the past 6 months**				0.833
No	300(33.9)	138(33.6)	162(34.2)	
Yes	584(66.1)	273(66.4)	311(65.8)	
**Condom use during the most recent anal sex**				0.049*
No	232(26.2)	95(23.1)	137(29.0)	
Yes	652(73.8)	316(76.9)	336(71.0)	
**Engagement in commercial sex with male in the past 6 months**				0.080
No	819(92.6)	374(91.0)	445(94.1)	
Yes	65(7.4)	37(9.0)	28(5.9)	
**Having sex with female in the past 6 months**				0.019*
No	717(81.1)	347(84.4)	370(78.2)	
Yes	167(18.9)	64(15.6)	103(21.8)	
**Sought sex via online platforms**				0.166
No	63(7.1)	24(5.8)	39(8.2)	
Yes	821(92.9)	387(94.2)	434(91.8)	
**STIs Characteristics**				
HIV testing				0.001*
Negative	821(92.9)	394(95.9)	427(90.3)	
Positive	63(7.1)	17(4.1)	46(9.7)	
**Chlamydia testing**				0.601
Negative	832(94.1)	385(93.7)	447(94.5)	
Positive	52(5.9)	26(6.3)	26(5.5)	
**Gonorrhea testing**				0.001*
Negative	846(95.7)	383(93.2)	463(97.9)	
Positive	38(4.3)	28(6.8)	10(2.1)	
**Syphilis testing**				0.039*
Negative	793(89.7)	378(92.0)	415(87.7)	
Positive	91(10.3)	33(8.0)	58(12.3)	
**Diagnosed with combined STIs in past year**				0.035*
No	829(93.8)	393(95.6)	436(92.2)	
Yes	55(6.2)	18(4.4)	37(7.8)	
**Awareness of using doxy-PEP**				
**Heard of doxy-PEP for STIs before**				0.104
No	657(74.3)	316(76.9)	341(72.1)	
Yes	227(25.7)	95(23.1)	132(27.9)	
**Previous received or are receiving doxy-PEP**				0.022*
No	848(95.9)	401(97.6)	447(94.5)	
Yes	36(4.1)	10(2.4)	26(5.5)	
**People around are receiving or have received doxy-PEP**				0.021*
No	743(84.0)	358(87.1)	385(81.4)	
Yes	141(16.0)	53(12.9)	88(15.6)	

* *P* < 0.05

### Sexual behaviors

Participants reported an average of 1.2 anal sex encounters with male partners in the past week (1.17 ± 1.58). In the last six months, 66.1% consistently used condoms, and 73.8% used a condom during their most recent encounter. About 7.4% engaged in commercial sex with male partners during this period, while 92.9% primarily sought partners online (Table 1).

### Prevalence of stis

The prevalence of HIV, chlamydia, gonorrhea, and syphilis among participants was 7.1%, 5.9%, 4.3%, and 10.3%, respectively, with a total STIs prevalence of 19.0% (Table 1).

### Awareness of Doxycycline post-exposure prophylaxis

Among participants, 227 (25.7%) were aware of doxy-PEP for STIs prevention before the survey. Nearly 5% had used or were currently using doxy-PEP, and 16.0% reported that someone in their social network had used it (Table 1).

### Willingness to use Doxycycline post-exposure prophylaxis

Approximately 53.5% of participants were open to using doxy-PEP for STIs prevention, with 76.7% primarily focused on preventing syphilis. However, 70.6% indicated they would not consistently use doxy-PEP after every high-risk encounter, and 75.1% anticipated no change in their condom use during anal sex. Additionally, over 90% would recommend doxy-PEP to others if shown to be effective (Table 2).

**Table 2:** Awareness of Doxycycline post-exposure prophylaxis among participants willing to use doxy-PEP (*N* = 473)

Variable	Total (*n*)	%
The preferring STIs to be prevented by using doxy-PEP		
Syphilis	363	76.7
Gonorrhea	268	56.7
Chlamydia	219	46.3
**Frequency of using doxy-PEP after high-risk sex if receiving doxy-PEP**		
Never	19	4.0
Sometime	153	32.3
Often	162	34.2
Always	139	29.4
**Changes in frequency of condom use during anal sex if receiving doxy-PEP**		
Increase frequency of condom use	61	12.9
As before	355	75.1
Decrease frequency of condom use	57	12.1
**The willingness to recommend doxy-PEP to peers if it is effective in preventing STIs**		
No	37	7.8
Yes	436	92.1

Of the 473 men interested in doxy-PEP, 83.9% preferred information from healthcare providers, such as doctors, nurses, or CDC officials. Secondary sources included the internet (45.4%), brochures or ads (41.6%), and peers (41.0%) (Table 3).

**Table 3:** Preferred methods of receiving information and primary concerns regarding doxy-PEP among participants willing to use it

Variable	Total (*n*)	%
The preferring form to receive the information about doxy-PEP (*N* = 473)		
Medical staff (doctors, nurses, CDC officials)	397	83.9
Internet	215	45.4
Educational brochure or advertising bar	197	41.6
Peers	194	41.0
Family members or friends	53	11.2
Newspaper/magazine	44	9.3
TV	38	8.0
**Primary concerns regarding using doxy-PEP (***N* **= 411)**		
Efficacy in preventing STIs	339	82.5
Safety	336	81.7
Drug resistance	295	71.8
Attitude of sexual partners	81	19.7
Endorsement or use of peers	24	5.8
Attitude of family	11	2.7
I don’t care at all	34	8.3

### Primary concerns regarding Doxycycline post-exposure prophylaxis

Most participants (82.5%) prioritized the “efficacy in preventing STIs” as their main concern regarding doxy-PEP, followed by safety (81.7%) and risk of drug resistance (71.8%). Fewer participants were concerned with sexual partners’ attitudes (19.7%), peer endorsement (5.8%), or family views (2.7%) (Table 3).

### Factors associated with willingness to use Doxycycline post-exposure prophylaxis

Multivariable logistic analysis showed that participants were more likely to express willingness to use doxy-PEP if they had female sexual partners in the past six months (aOR = 1.58, 95% CI: 1.11–2.25), tested positive for HIV (aOR = 2.55, 95% CI: 1.42–4.57) or syphilis (aOR = 1.58, 95% CI: 1.00–2.49), had prior or current doxy-PEP use (aOR = 2.39, 95% CI: 1.13–5.05), or had peers currently using doxy-PEP (aOR = 1.58, 95% CI: 1.09–2.30) (Table 4).

**Table 4:** Factors associated with willingness to use Doxycycline post-exposure prophylaxis among MSM (*N* = 884)

Characteristics	cOR(95% CI)	*P*-value	aOR(95% CI) ^a^	*P*-value
**Demographics**				
** Age**				
18–25	*Ref*		*Ref*	
26–35	1.35(0.93–1.95)	0.117	1.36(0.93–1.98)	0.112
36–45	1.01(0.66–1.54)	0.962	1.03(0.66–1.61)	0.903
>45	0.86(0.57–1.28)	0.453	0.90(0.57–1.44)	0.673
** Ethnicity**				
Han	*Ref*		*Ref*	
Non-Han	0.98(0.55–1.72)	0.934	0.91(0.51–1.63)	0.743
** Marital status**				
Unmarried/Devoice	*Ref*		*Ref*	
Married	0.82(0.60–1.11)	0.187	0.91(0.64–1.30)	0.600
** Place of residence**				
Local province	*Ref*		*Ref*	
Other province	1.23(0.93–1.62)	0.145	1.42(1.05–1.92)	0.022*
** Educational attainment**				
Junior high school or below	*Ref*		*Ref*	
High school	0.85(0.59–1.22)	0.368	0.83(0.57–1.21)	0.332
College degree or above	1.27(0.89–1.82)	0.182	1.22(0.82–1.81)	0.319
** Duration of employment at the current location**				
< 6 months	*Ref*		*Ref*	
6–12 months	1.19(0.66–2.14)	0.57	1.24(0.68–2.24)	0.483
Over 1 year	1.31(0.89–1.94)	0.18	1.36(0.91–2.06)	0.138
** Sexual orientation**				
Homosexual	*Ref*		*Ref*	
Bisexual	1.08(0.80–1.48)	0.61	1.08(0.79–1.49)	0.622
Other	1.58(0.96–2.59)	0.069	1.64(0.99–2.70)	0.054
** Received any of the AIDS prevention services in the past year**				
No	*Ref*		*Ref*	
Yes	1.68(0.84–3.36)	0.146	1.52(0.75–3.07)	0.243
** Sexual behavior**				
** Number of anal sex with male in the past week**				
None	*Ref*		*Ref*	
1–3	0.74(0.56–0.97)	0.028*	0.78(0.59–1.04)	0.087
>3	0.41(0.23–0.72)	0.002*	0.45(0.26–0.80)	0.007*
** Providing anal sex for males in the past 6 months**				
No	*Ref*		*Ref*	
Yes	0.73(0.42–1.27)	0.265	0.70(0.40–1.23)	0.215
** Consistent condom uses during anal sex with males in the past 6 months**				
No	*Ref*		*Ref*	
Yes	0.97(0.73–1.28)	0.833	0.92(0.70–1.23)	0.591
** Used condom at last anal sex**				
No	*Ref*		*Ref*	
Yes	0.74(0.54-1.00)	0.049*	0.73(0.54-1.00)	0.05*
** Sought sex via online platforms**				
No	*Ref*		*Ref*	
Yes	0.69(0.41–1.17)	0.167	1.23(0.84–1.82)	0.286
** Looking for commercial sex with male in the past 6 months**				
No	*Ref*		*Ref*	
Yes	0.64(0.38–1.06)	0.082	0.72(0.43–1.21)	0.219
** Having sex with female in the past 6 months**				
No	*Ref*		*Ref*	
Yes	1.51(1.07–2.13)	0.019*	1.58(1.11–2.25)	0.011*
**STIs Characteristics**				
** HIV testing results**				
Negative	*Ref*		*Ref*	
Positive	2.50(1.41–4.43)	0.002*	2.55(1.42–4.57)	0.002*
** Chlamydia testing results**				
Negative	*Ref*		*Ref*	
Positive	0.86(0.49–1.51)	0.602	0.88(0.50–1.54)	0.664
** Gonorrhea testing results**				
Negative	*Ref*		*Ref*	
Positive	0.30(0.14–0.62)	0.001*	0.33(0.16–0.70)	0.004*
** Syphilis testing results**				
Negative	*Ref*		*Ref*	
Positive	1.60(1.02–2.51)	0.040*	1.58(1.00-2.49)	0.049*
** Diagnosed with combined STIs in past year**				
No	*Ref*		*Ref*	
Yes	1.85(1.04–3.31)	0.037*	1.80(1.00-3.24)	0.051
**Awareness of using doxy-PEP**				
** Heard of doxy-PEP for STIs before**				
No	*Ref*		*Ref*	
Yes	1.29(0.95–1.75)	0.104	1.29(0.94–1.75)	0.111
** Previous received or are receiving doxy-PEP**				
No	*Ref*		*Ref*	
Yes	2.33(1.11–4.90)	0.025*	2.39(1.13–5.05)	0.022*
** Peers or sexual partners around are undergoing or have undergone doxy-PEP**				
No	*Ref*		*Ref*	
Yes	1.54(1.07–2.24)	0.021*	1.58(1.09–2.30)	0.016*

* *P* < 0.05

^a^Age, ethnicity, marital status, education level, household registration and length of time working in the current location were adjusted for each other, all other variables were adjusted for age, ethnicity, marital status, education level, monthly income, household registration and length of time working in the current location

## Discussion

Doxy-PEP presents a promising approach for STI prevention among MSM. In our study, over half of participants indicated a willingness to use doxy-PEP, offering important insights into factors affecting its acceptance and highlighting common concerns among hesitant users. These findings can guide doxy-PEP implementation and expansion efforts for MSM in China.

Our study reported a 53.5% willingness to use doxy-PEP among MSM in southern China, lower than rates observed in Canada (60.1%) [20], the United States (67.5%) [21], and one prior study in Chinese MSM (74.8%) [13]. Participants’ primary concerns centered on doubts about doxy-PEP’ s efficacy and safety, consistent with barriers identified in previous studies [13, 22], which suggests that addressing these issues could improve acceptance. Despite these reservations, multiple randomized controlled trials confirm doxy-PEP’ s strong efficacy and safety profile in preventing STIs [23]. Guidelines supporting doxy-PEP use have been established in countries including the United States [11], Australia [24], and several European nations [25], yet remain absent in China. In the U.S, MSM willingness to use doxy-PEP increased significantly following CDC guideline publication [26]. Given China’ s high STIs prevalence, developing national guidelines or expert consensus could improve doxy-PEP acceptance among Chinese MSM.

We also found that most participants faced challenges in maintaining adherence to doxy-PEP, even when it was available. Adherence to doxy-PEP, defined as taking a 200 mg dose of doxycycline within 72 h of condomless sex, is essential for its effectiveness, and prior studies indicate that low adherence substantially reduces its protective benefits [27]. Common factors contributing to low adherence include efficacy and safety concerns, limited awareness, forgetfulness, inconvenience, medication stigma, and perceived low STIs risk [28–30]. Insights from HIV pre-exposure prophylaxis (PrEP) research, such as targeted health education, automated reminders, and remote support, have proven effective in enhancing adherence [31–34]. Given the central role of adherence in doxy-PEP effectiveness, future research should focus on identifying adherence barriers and developing targeted strategies to improve doxy-PEP adherence among MSM.

Our study found that participants were more likely to use doxy-PEP if friends or sexual partners had also used it, indicating that social networks could play a critical role in promoting doxy-PEP awareness and acceptance. This observation aligns with prior research on Chinese MSM [13], suggesting that peer influence can positively impact health behavior adoption. First, individuals may better understand prevention methods by learning from peers with similar risk profiles [35], which helps alleviate concerns about doxy-PEP use. Second, as doxy-PEP becomes more accepted within the community, stigma around its use may lessen, further encouraging uptake [36]. Previous studies have shown that peer-based health information sharing supports the adoption of health practices, such as HIV [37] and syphilis [38] self-testing among MSM. Additionally, nearly all participants in our study reported a willingness to recommend doxy-PEP to peers, underscoring the potential effectiveness of peer-based strategies to increase doxy-PEP uptake among MSM. Future implementation efforts should harness social networks to expand doxy-PEP outreach within this community.

This study has several limitations. First, the cross-sectional design limits causal inference between predictors and willingness to use doxy-PEP, necessitating future longitudinal studies. Second, self-reported data may introduce recall and social desirability biases, particularly regarding risky sexual behaviors. Third, previous research indicates that younger MSM are at higher risk for STIs [39, 40]. However, our study included a relatively small proportion of younger MSM, which may limit the generalizability of our findings to this group. Fourth, the venue-based convenience sampling in four cities of Guangdong Province introduces significant selection bias and limits external validity, particularly for MSM who don’t frequent these venues or reside in rural areas. Fifth, our definition of syphilis positivity does not distinguish between newly acquired and previous infections, which may lead to an overestimation of the prevalence of syphilis. Finally, while our sample size was substantial, the non-random recruitment from cities with extensive outreach may further constrain the generalizability of results to other urban settings.

## Conclusion

In conclusion, our study found that 53.5% of MSM were willing to adopt doxy-PEP, indicating a moderate level of acceptance that may vary by region or population. As further trials confirm its effectiveness in STIs prevention, expanding doxy-PEP’s use among MSM should be prioritized. Establishing clear guidelines or expert consensus in China will be essential to improve its acceptance and adoption as a preventive measure in this group.

## Data Availability

The datasets used and/or analyzed during the current study are available from the corresponding author on reasonable request.

## Supplementary Infornation

Supplementary Material 1.

## Abbreviations

MSM: Men who have Sex with Men
STIs: Sexually Transmitted Infections
Doxy-PEP: Doxycycline post-exposure prophylaxis
CI: Confidence interval
aOR: Adjusted Odds Ratio
CDC: Centers for Disease Control and Prevention
US: the United States
HIV: Human Immunodeficiency Virus
