# Supplementary material for: Willingness to accept Doxycycline post-exposure prophylaxis for bacterial stis prevention among men who have sex with men in Southern China: a cross-sectional analysis
Source: BMC Infect Dis. 2025 Jul 11;25:911. doi: 10.1186/s12879-025-11290-x (PMC12247278; doi:10.1186/s12879-025-11290-x)
Supplement: Supplementary file 1 — Supplementary Material 1. [file 12879_2025_11290_MOESM1_ESM.docx]

# Additional file 1: Willingness to use Doxycycline Post-Exposure Prophylaxis for Bacterial STIs Prevention Among MSM in Guangdong province, survey

Hello, my name is... from…. We are conducting a survey to find out what people know and do about a number of health issues. Please be assured that this survey is anonymous and your responses will be kept confidential. We hope that your answer is your real personal situation. The survey will take about 10 minutes of your time, and at the end of the survey I can offer you some help (for example, you can ask some health questions and I will try to answer them). We hope you will support our work. Thank you!

Ask the respondents:

① Would you like to participate in this survey? If the answer is "no", the visit will be terminated;

② Have you taken part in this survey recently? If the answer is "yes", the visit will end.

1. Birth Year

______

1. Ethnicity
   1. Han
   2. Other:
2. Marriage with opposite sex
   1. Never married
   2. At marriage
   3. Divorced
3. Household registration
   1. Guangdong
   2. Other province:
4. Educational level
   1. Junior high school or below
   2. High school or Dazhuan
   3. High school or above
5. Length of time living in current location
   1. Less than 3 months
   2. 3~6 months
   3. 7~12 months
   4. 1~2 years
   5. More than 2 years
6. What is your sexual orientation?
   1. Homosexuality
   2. Bisexuality
   3. Don’t know
7. In the past year, have you received any of the services related to HIV prevention (including condom promotion, HIV counseling and testing, community drug maintenance therapy, exchange of clean needles or peer education)
   1. Yes
   2. No
8. In the last six months, have you had anal sex with someone of the same sex?
   1. Yes
   2. No
9. In the last week, how many times have you had anal sex with someone of the same sex?

_______

1. In the last six months, how often have you used condoms when having anal sex with someone of the same sex?
   1. Never used
   2. Sometimes used
   3. Every time
2. In the last six months, did you use a condom the last time you had anal sex with someone of the same sex?
   1. Yes
   2. No
3. In the last six months, have you had commercial sex with someone of the same sex?
   1. Yes
   2. No
4. Have you had sex with the female in the last six months?
   1. Yes
   2. No
5. Have you ever looked for sexual partners via online platforms?
   1. Yes
   2. No
6. Have you ever been diagnosed with any STIs (gonorrhea, syphilis and chlamydia) in the last year?
   1. Yes
   2. No

| ****Public Health Information:**** Post-exposure prophylaxis (doxy-PEP) for bacterial sexually transmitted deceases (STIs) involves taking antibiotics after each high-risk sexual encounter to prevent bacterial sexually transmitted infections. The efficacy of doxy-PEP in preventing STIs has been demonstrated in multiple countries. |
| --- |

1. Have you ever heard of doxycycline post-exposure prophylaxis (doxy-PEP) for bacterial STIs (chlamydia, gonorrhea, or syphilis)?
   1. Yes
   2. No (Skip to E1)
2. Have you received or are you currently receiving doxy-PEP for bacterial STIs?
   1. Yes
   2. No
3. Do you know anyone around you who is currently receiving or has received doxy-PEP?
   1. Yes
   2. No
   3. I’m not sure
4. Are you willing to receive doxy-PEP for bacterial STIs?
   1. Yes
   2. No
5. If you choose doxy-PEP which infection are you more inclined to prevent? (multiple options)
   1. Chlamydia
   2. Syphilis
   3. Gonorrhea
   4. Other _____
6. If you receive doxy-PEP for bacterial STIs, how likely are you to take the medication after each high-risk sexual encounter?
   1. Every time
   2. Most of the time
   3. Sometimes
   4. Never
7. If you receive doxy-PEP, how do you think your frequency of using condoms during sexual encounters with others will change?
   1. Increase
   2. Stay the same as before
   3. Decrease
8. What concerns you most about doxy-PEP for preventing bacterial STIs (chlamydia, gonorrhea, syphilis)? (multiple options)
   1. Safety (e.g., nausea, gastrointestinal discomfort, etc.)
   2. Drug resistance
   3. Effectiveness of prevention
   4. Support from sexual partners
   5. Support from family
   6. Whether peers are using it
   7. I don’t care at all
9. If doxy-PEP is effective in preventing bacterial STIs, would you recommend it to people around you?
   1. Yes
   2. No
10. Through which channels would you like to learn about doxy-PEP information for bacterial STIs? (multiple options)
    1. Internet
    2. TV
    3. Newspaper/magazine
    4. Educational brochure or advertising bar
    5. Peers
    6. Medical staff (doctors, nurses, CDC officials)
    7. Family members or friends

This investigation is over. Thank you for your cooperation. To know your health status, we need to collect your blood and test for HIV and syphilis. Urine swabs were collected from you to test for gonorrhea and chlamydia.

1. Whether urine was taken for this investigation?
   1. Yes
   2. No
2. Urine chlamydia test results
   1. Positive
   2. Negative
   3. Specimen invalid (fill in according to the feedback result of laboratory)
3. Urine gonococcal test results
   1. Positive
   2. Negative
   3. Specimen invalid (fill in according to the feedback result of laboratory)

E4. Whether anal swab was taken for this investigation?

- 1. Yes
  2. No

E5. Anal swab chlamydia test results

- 1. Positive
  2. Negative
  3. Specimen invalid (fill in according to the feedback result of laboratory)

E6. Anal swab gonococcal test results

- 1. Positive
  2. Negative
  3. Specimen invalid (fill in according to the feedback result of laboratory)

E8. Whether oral swab was taken for this investigation?

- 1. Yes
  2. No

E9. Oral swab chlamydia test results

- 1. Positive
  2. Negative
  3. Specimen invalid (fill in according to the feedback result of laboratory)

E10. Oral swab gonococcal test results

- 1. Positive
  2. Negative
  3. Specimen invalid (fill in according to the feedback result of laboratory)

E11. Whether blood was taken for this investigation?

aYes

bNo

E12. HIV antibody test results:

First ELISA preliminary screening:

- 1. Positive
  2. Negative

Second ELISA test:

- 1. Positive
  2. Negative

Confirmed test

- 1. Positive
  2. Negative
  3. Suspicious
  4. Untested

E13. Syphilis test results Whether blood was taken for this investigation?

ELISA test:

- 1. Positive
  2. Negative (ending)

RPR/TRIST tests:

- 1. Positive
  2. Negative
